# Characteristic analysis of adverse reactions of finerenone: an in-depth analysis from WHO-VigiAccess

**DOI:** 10.3389/fphar.2025.1545148

**Published:** 2025-08-07

**Authors:** Hongxuan Fan, Zhuolin Huang, Yafen Yang, Jiahui Li, Boda Zhou

**Affiliations:** ^1^ Department of Cardiology, Beijing Tsinghua Changgung Hospital, School of Clinical Medicine, Tsinghua University, Beijing, China; ^2^ Department of Cardiology, The Second Hospital of Shanxi Medical University, Taiyuan, Shanxi, China

**Keywords:** finerenone, VigiAccess, adverse events, mineralocorticoid receptor antagonist, drug safety

## Abstract

**Introduction:**

Finerenone is a novel non-steroidal mineralocorticoid receptor antagonist that has shown promise in the treatment of chronic kidney disease and heart failure. As its clinical use expands, understanding the adverse events associated with finerenone becomes crucial to ensuring patient safety. Prior pharmacovigilance studies have not systematically mapped finerenone-related AEs across all organ systems using global spontaneous-reporting data. We therefore aimed to identify and quantify these signals in the WHO-VigiAccess database.

**Methods:**

This study employed a retrospective descriptive analysis using the reporting odds ratio (ROR), proportional reporting ratio (PRR), bayesian confidence propagation neural network (BCPNN) and empirical bayes geometric mean (EBGM) approaches to investigate reports of AEs associated with finerenone. Data were sourced from WHO’s VigiAccess database, focusing on affected organ systems, symptoms, and demographic details, such as age, gender, and geographic distribution of the patients in the reports. The VigiAccess database was queried in November 2024 to collect data on AEsreported after the administration of finerenone.

**Results:**

A total of 1,482 AEs associated with finerenone were reported in VigiAccess by the end of November 2024. The analysis identified the five most frequently reported AEs, including hyperkalaemia (N = 272, ROR = 244.39), glomerular filtration rate dereased (N = 186, ROR = 684.34), blood potassium increased (N = 141, ROR = 372.63), blood creatinine increased (N = 100, ROR = 50.89), death (N = 62, ROR = 3.28), hypotension (N = 46, ROR = 5.45). The five most common categories of AEs included investigations yielding undesirable outcomes (636 cases, 26.67%), metabolism and nutrition disorders (360 cases, 15.09%), general disorders and administration site conditions (263 cases, 11.03%), gastrointestinal disorders (211 cases, 8.85%), renal and urinary disorders (159 cases, 6.67%).

**Conclusion:**

The study highlighted the significance of monitoring AEsrelated to finerenone, with 1,482 AEs reported by November 2024. While many AEs were mild and self-limiting, some were severe, potentially leading to hospitalization or serious health implications. It is imperative for healthcare systems to engage in robust safety research and monitoring to better understand the causal relationships between finerenone and reported AEs, ensuring safer therapeutic outcomes for patients.

## Introduction

CKD is a significant global health issue characterized by the gradual loss of kidney function, potentially leading to end-stage renal disease (Kalantar-Zadeh et al., 2021). Severe proteinuria is a key clinical manifestation of CKD, indicating kidney damage and a poor prognosis (Tarun et al., 2024). For patients, persistent protein excretion not only exacerbates the burden on the kidneys but is also closely associated with an increased risk of cardiovascular diseases, leading to a significant decline in quality of life and high medical costs. Therefore, understanding and managing proteinuria in CKD is crucial for alleviating the patient’s burden (Wang et al., 2023).

MRA is a category of pharmaceuticals that inhibit the effects of aldosterone, a hormone that enhances salt reabsorption and potassium excretion in the kidneys. This activity is fundamental to the body’s mechanisms for regulating blood pressure and fluid equilibrium (Kintscher et al., 2022). By inhibiting these receptors, MRAs diminish sodium reabsorption and enhance potassium retention, so aiding in the management of diseases such as hypertension, heart failure, and specific forms of CKD.

There are two primary categories of MRAs: steroidal (e.g., spironolactone and eplerenone) and non-steroidal (e.g., finerenone). Steroidal MRAs have been utilized for decades and are recognized for their efficacy; nonetheless, they may induce side effects owing to their interaction with other steroid hormone receptors. Non-steroidal MRAs, such as finerenone, are engineered to deliver comparable therapeutic advantages while potentially minimizing adverse effects due to their enhanced selectivity for the mineralocorticoid receptor (Agarwal et al., 2021). MRAs have demonstrated considerable advantages in mitigating cardiovascular and renal risks, especially in individuals with diseases such as heart failure with reduced ejection fraction or diabetic nephropathy. Nonetheless, their mechanism of action presents a danger of hyperkalemia (elevated potassium levels), necessitating vigilant monitoring during treatment (Barrera-Chimal et al., 2022). MRAs are essential in managing intricate disorders impacting cardiovascular and renal health, underscoring the necessity for personalized treatment strategies and regular monitoring to enhance therapeutic results while mitigating hazards (Barrera-Chimal et al., 2019).

Recent research has demonstrated that finerenone has significant pharmacological effects, particularly offering cardiovascular and renal benefits (González-Juanatey et al., 2023; Solomon et al., 2024; Agarwal et al., 2022a). Specifically targeting the mineralocorticoid receptor is the key mechanism of action, which is responsible for efficiently reducing the hyperactivity of the renin-angiotensin-aldosterone system. Considering that this method significantly lessens the amount of microalbumin that is excreted in the urine, it is widely utilized in the treatment of individuals who suffer from CKD and diabetic nephropathy (Bakris et al., 2020). Compared to previous generations of medications, finerenone shows greater selectivity for mineralocorticoid receptors, which helps reduce adverse effects such as hyperkalemia associated with older drugs. Nonetheless, monitoring for hyperkalemia remains crucial. Additionally, there are some limitations, including the need for ongoing assessment of long-term safety data in clinical applications. The utilization of finerenone in the therapy of CKD is strongly advocated by recent international guidelines, including KDIGO 2024 (Kidney Disease: Improving Global Outcomes KDIGO CKD Work Group, 2024). This is mainly due to the endorsement of pivotal randomized controlled studies, which further support the utilization of finerenone. The findings of many studies, such as the FIDELIO-DKD (Agarwal et al., 2022b) and FIGARO-DKD (Filippatos et al., 2022a) trials, highlight the significant therapeutic benefits that it offers in preventing the progression of renal disease and cardiovascular events. The results of these investigations provide strong evidence that finerenone has the ability to significantly reduce the number of clinical endpoints that occur.

It is imperative that the potential adverse effects of finerenone not be ignored, despite the fact that it possesses obvious benefit in therapeutic settings (Zhang et al., 2022). Because of the growing number of users and the expanding range of indications, the monitoring of AEsis becoming an increasingly important aspect of the process (Docherty et al., 2024). Safety studies that make use of data from the real world are especially important because of the limitations that are inherent to conventional randomized controlled trials that are conducted with very selective cohorts. Even if they have some limitations, spontaneous reporting systems provide a substantial perspective that can be utilized for assessing the safety of finerenone in real-world settings (Luo et al., 2022). This study, which is based on data collected from the actual world, contributes to the identification and comprehension of potential AEs that may occur throughout a larger population. As a result, it provides a more comprehensive guarantee of the safety of drugs.

However, the recorded side effects highlight the requirement of emphasizing safety in their application. It is crucial that we improve our understanding of the harmful effects of finerenone in order to ensure that they continue to be safe and acceptable in the future (Huang et al., 2024). For the purpose of optimizing therapeutic results and enhancing public health advantages, these specialists in the healthcare industry need to adapt treatment programs according to the risk profiles of individual patients. We will be able to protect the health and welfare of patients in a more efficient manner if we continue to improve and refine the protocols that are used for medical safety monitoring. Despite the growing clinical uptake of finerenone, the existing literature lacks a comprehensive, population-level evaluation of its adverse-event spectrum across all organ systems, with most reports limited to controlled trial settings or single-organ outcomes. This evidence gap undermines risk-benefit assessments in routine care. Leveraging the global coverage of the WHO-VigiAccess database, the present study was designed to systematically detect, quantify, and describe finerenone-related adverse-event signals to guide safer therapeutic use.

## Methods

### Search strategy and data source

An inquiry by WHO-VigiAccess was conducted on 24 November 2024 to identify recorded adverse occurrences following the use of finerenone. The user login webpage is https://www.vigiaccess.org. The WHO-VigiAccess compiles global statistics on age demographics, gender, reporting year, and continents (van De Ven et al., 2020). Uppsala monitoring center (UMC) can acquire pharmaceutical safety records using the complimentary programme for international drug monitoring (PIDM) database interface, WHO-VigiAccess. VigiAccess is a public resource that provides a statistical summary of the data included in VigiBase. The VigiBase database was established in 1968 when data were provided by member nations of the International Drug Monitoring Programme, initially comprising only 10 countries. As of March 2022, it consists of 155 full member nations and 21 associate member states awaiting approval for full membership. ICSRs submitted by member states are predominantly reported by healthcare professionals, patients, and pharmaceutical companies to their national drug regulatory authorities, which are then analyzed and aggregated before being forwarded to VigiBase. The definition is based on the Preferred Terms (PTs) and System Organ Class (SOC) from the Medical Dictionary for Regulatory Activities (MedDRA). To outline the toxicity spectrum, data for each medication were gathered, and each adverse event was classified using the MedDRA SOC and PT classifications documented. Multiple dictionaries, including the World Health Organization Adverse Reaction Terminology (WHO-ART) (Edwards and Aronson, 2000), provide the reporting terminology employed in MedDRA. Twenty of the 27 items in the SOC category directly related to disease symptoms were chosen for analysis. The pharmaceutical name serves as the search criterion, while the active ingredients are assessed to attain the desired outcome.

### Disproportionality analysis

Through disproportionality analysis, we applied four methods for disproportionate reporting: the reporting odds ratio (ROR) (Rothman et al., 2004) and the proportional reporting ratio (PRR) (Evans et al., 2001). The calculation of ROR and PRR depends on the evaluation of odds disparity, a method commonly utilized in adverse event signal detection (van Puijenbroek et al., 2002). The ROR is calculated to evaluate the probability difference of reporting an adverse event (AE) for a certain medication compared to other drugs. The equation produces the reporting odds ratio (ROR):

$$ROR=\frac{a\times d}{b\times c}$$

The data in (a), (b), (c), and (d) represent the number of reports related to the specific drug and specific AE, the number of reports for the specific drug and other AEs, and the number of reports for other drugs and other AEs. A minimum of three cases (a ≥3) is necessary for each medication and AE combination to guarantee the statistical robustness of the ROR computation. The PRR is an additional metric for evaluating the disproportionality of AE reports. It is calculated as:

$$PRR=\frac{a\left(c+d\right)}{c\left(a+b\right)}$$

Similar to ROR, for the specific drug and AE combination (a ≥3) to be considered valid, the PRR calculation requires at least three examples. If both the ROR number and the lower limit of the 95% Confidence Interval (CI) for ROR exceed 2 (ROR > 2) and 1 (Lower limit of 95% CI of ROR > 1), the signal is considered disproportionate and may indicate a safety issue. These criteria guarantee that the observed disproportionality is not attributable to random variability. Applying ROR, PRR, BCPNN and EBGM in our study, we conducted a comprehensive assessment of the disproportionality of AEs associated with finerenone. The analysis results support pharmacovigilance strategies aimed at enhancing drug safety. BCPNN (Zou et al., 2024) and EBGM (Zhang et al., 2024) methods were included and described in Supplementary Table S1.

### Statistical analysis

The study utilised a retrospective quantitative design. Descriptive analysis in Excel was utilised to investigate the attributes of victims exhibiting adverse reactions to AEs associated with finerenone. The ADR report rate was calculated as the ratio of ADR symptoms linked to AEs to the overall number of ADR complaints. The prevalent ADRs relate to the symptoms linked to the top 36 ADR report frequencies. The frequency of reported ADR symptoms was calculated, and a descriptive comparative analysis was performed. Frequencies and percentages were utilised to categorise descriptive variables.

## Results

### Description of the studied cases

**FIGURE 1:**
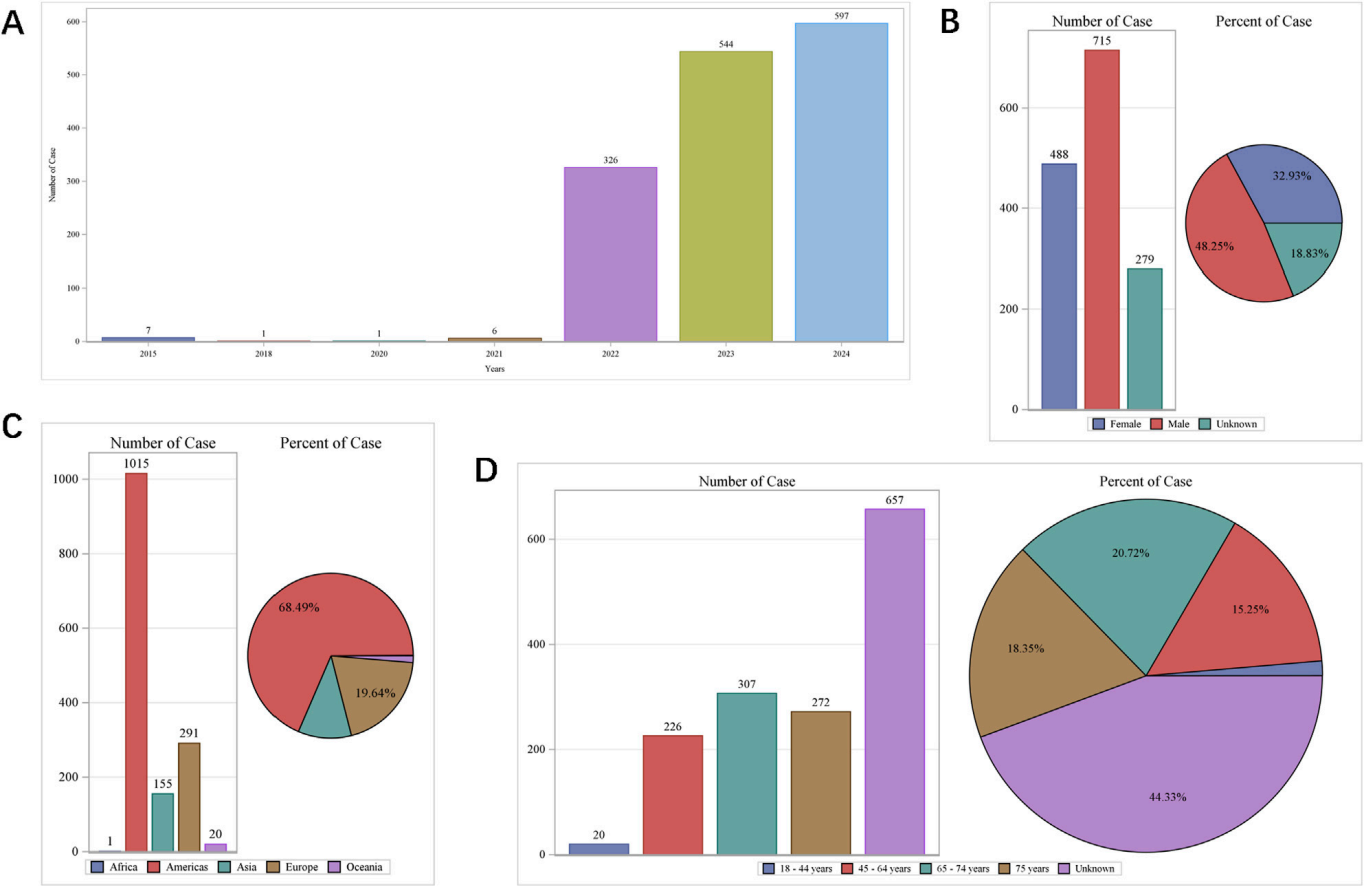
Baseline characteristics of participants self-reporting adverse events following finerenone usage. **(A)** Distribution of cases by year; **(B)** Gender distribution of self-reported cases; **(C)** Geographic distribution across continents; **(D)** Age group distribution.

The earliest reports of AEs associated with finerenone were received in the WHO-VigiAccess database in 2015. It was not until 2022 that reports of its AEs began to emerge in significant numbers. In 2022, there were 326 reported cases of AEs associated with finerenone. By November 2024, the WHO had received a total of 1,482 AEs associated with finerenone. The number of AEs increased in 2023, reached notably high at 544 cases. In 2024, there was a considerable increase with the cases expected to increase to over 600 (Figure 1A). Figure 1B illustrates that AEs were more prevalent among male recipients, with males accounting for approximately half of cases. Among the continents, Americas reported the highest number of AEs, while Africa reported the lowest, as shown in Figure 1C. Age-wise, AEs were most frequent in the 65–74 age group, with 307 cases, and least common among finerenone recipients under 45 years of age, as depicted in Figure 1D.

### Distribution of top 12 SOCs and PTs of AEs associated with finerenone

**FIGURE 2:**
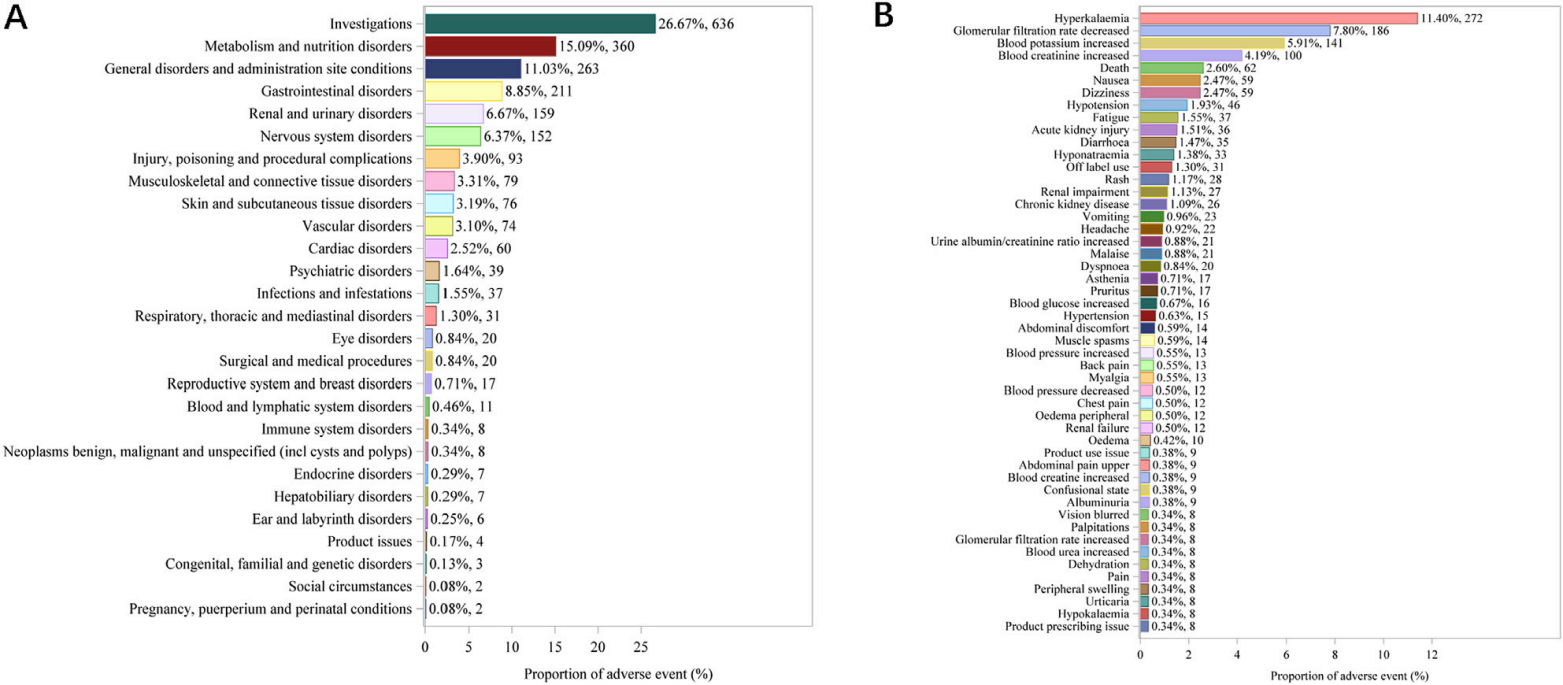
Top 27 SOCs **(A)** and top 50 PTs **(B)** of adverse events following finerenone usage from the VigiAccess database, presented as percentages and counts.

The top ten commonly reported AEs associated with system organ class (SOC) are as follows (Figure 2A): investigations yielding undesirable outcomes (636 cases, 26.67%), metabolism and nutrition disorders (360 cases, 15.09%), general disorders and administration site conditions (263 cases, 11.03%), gastrointestinal disorders (211 cases, 8.85%), renal and urinary disorders (159 cases, 6.67%), nervous system disorders (152 cases, 2.13%), injury, poisoning and procedural complications (93 cases, 3.90%), musculoskeletal and connective tissue disorders (79 cases, 3.31%), skin and subcutaneous tissue disorders (76 cases, 3.19%), vascular disorders (74 cases, 3.10%), cardiac disorders (60 cases, 2.52%) and psychiatric disorders (39 cases, 1.64%). Additionally, the twelve most frequently reported AEs manifestations include hyperkalaemia, glomerular filtration rate dereased, blood potassium increased, blood creatinine increased, death, nausea, dizziness, hypotension, fatigue, acute kidney injury, diarrhoea and hyponatraemia (Figure 2B). These manifestations are the PTs within their respective SOC categories. While most of these commonly reported AEs are minor and self-limiting, there are some serious AEs, such as hyperkalaemia (272 cases, 11.40%), blood potassium increased (141 cases, 5.91%), death (62 cases, 2.60%), dizziness (59 cases, 2.49%), hypotension (46 cases, 1.93%).

### Signal detection at SOC level tested by four methods

After a combination of PRR, ROR, PRR, BCPNN, and EBGM, both known for their heightened sensitivity, were utilized in our signal mining process. The results of four analysis methods of 27 SOCs were displayed in Table 1. The top 12 commonly reported AEs (and this order is consistent with their frequency) associated with SOC are as follows: investigations yielding undesirable outcomes (N = 636, ROR = 5.39), metabolism and nutrition disorders (N = 360, ROR = 9.76), general disorders and administration site conditions (N = 263, ROR = 0.49), gastrointestinal disorders (N = 211, ROR = 0.88), renal and urinary disorders (N = 159, ROR = 4.39), nervous system disorders (N = 152, ROR = 0.60), injury, poisoning and procedural complications (N = 93, ROR = 0.59), musculoskeletal and connective tissue disorders (N = 79, ROR = 0.62), skin and subcutaneous tissue disorders (N = 76, ROR = 0.34), vascular disorders (N = 74, ROR = 1.47), cardiac disorders (N = 60, ROR = 1.06) and psychiatric disorders (N = 39, ROR = 0.33).

**TABLE 1:** Signal strength of ADEs at the System Organ Class (SOC) level in Vigiaccess database.

System organ Class (SOC)	SOC code	Case reports	ROR (95% CI)	PRR (95% CI)	Chi_Square	IC(IC025)	EBGM(EBGM05)
Investigations	10022891	636	5.39 (4.92, 5.90)	4.22 (3.95, 4.51)	1, 668.15	2.08 (1.94)	4.22 (3.85)
Metabolism and nutrition disorders	10027433	360	9.76 (8.73, 10.92)	8.44 (7.67, 9.28)	2403.80	3.08 (2.88)	8.44 (7.54)
General disorders and administration site conditions	10018065	263	0.49 (0.43, 0.55)	0.54 (0.49, 0.61)	126.35	−0.88 (−1.06)	0.54 (0.48)
Gastrointestinal disorders	10017947	211	0.88 (0.77, 1.02)	0.89 (0.78, 1.02)	3.04	−0.16 (−0.37)	0.89 (0.77)
Renal and urinary disorders	10038359	159	4.39 (3.73, 5.15)	4.16 (3.58, 4.83)	387.92	2.06 (1.79)	4.16 (3.54)
Nervous system disorders	10029205	152	0.60 (0.51, 0.70)	0.62 (0.53, 0.73)	38.85	−0.68 (−0.92)	0.62 (0.53)
Injury, poisoning and procedural complications	10022117	93	0.59 (0.48, 0.73)	0.61 (0.50, 0.74)	25.09	−0.72 (−1.02)	0.61 (0.49)
Musculoskeletal and connective tissue disorders	10028395	79	0.62 (0.49, 0.77)	0.63 (0.51, 0.78)	18.27	−0.67 (−0.99)	0.63 (0.50)
Skin and subcutaneous tissue disorders	10040785	76	0.34 (0.27, 0.43)	0.36 (0.29, 0.45)	94.25	−1.47 (−1.79)	0.36 (0.29)
Vascular disorders	10047065	74	1.47 (1.17, 1.85)	1.46 (1.16, 1.82)	10.77	0.54 (0.19)	1.46 (1.15)
Cardiac disorders	10007541	60	1.06 (0.82, 1.37)	1.06 (0.83, 1.36)	0.21	0.08 (−0.29)	1.06 (0.82)
Psychiatric disorders	10037175	39	0.33 (0.24, 0.46)	0.34 (0.25, 0.47)	51.04	−1.54 (−1.97)	0.34 (0.25)
Infections and infestations	10021881	37	0.38 (0.27, 0.52)	0.39 (0.28, 0.53)	37.45	−1.37 (−1.82)	0.39 (0.28)
Respiratory, thoracic and mediastinal disorders	10038738	31	0.29 (0.20, 0.41)	0.30 (0.21, 0.42)	54.44	−1.76 (−2.24)	0.30 (0.21)
Surgical and medical procedures	10042613	20	1.03 (0.67, 1.60)	1.03 (0.67, 1.60)	0.02	0.05 (−0.59)	1.03 (0.67)
Eye disorders	10015919	20	0.48 (0.31, 0.75)	0.49 (0.31, 0.75)	11.08	−1.04 (−1.64)	0.49 (0.31)
Reproductive system and breast disorders	10038604	17	0.67 (0.41, 1.07)	0.67 (0.42, 1.07)	2.84	−0.58 (−1.24)	0.67 (0.41)
Blood and lymphatic system disorders	10005329	11	0.21 (0.11, 0.37)	0.21 (0.12, 0.38)	33.72	−2.26 (−3.00)	0.21 (0.12)
Neoplasms benign, malignant and unspecified	10029104	8	0.24 (0.12, 0.48)	0.24 (0.12, 0.48)	19.50	−2.06 (−2.89)	0.24 (0.12)
Immune system disorders	10021428	8	0.26 (0.13, 0.53)	0.27 (0.13, 0.53)	16.30	−1.91 (−2.75)	0.27 (0.13)
Hepatobiliary disorders	10019805	7	0.37 (0.17, 0.77)	0.37 (0.18, 0.77)	7.64	−1.44 (−2.34)	0.37 (0.18)
Endocrine disorders	10014698	7	1.46 (0.70, 3.07)	1.46 (0.70, 3.06)	1.02	0.55 (−0.56)	1.46 (0.70)
Ear and labyrinth disorders	10013993	6	0.47 (0.21, 1.04)	0.47 (0.21, 1.04)	3.68	−1.10 (−2.08)	0.47 (0.21)
Product issues	10077536	4	0.18 (0.07, 0.49)	0.19 (0.07, 0.50)	14.36	−2.43 (−3.46)	0.19 (0.07)
Congenital, familial and genetic disorders	10010331	3	0.74 (0.24, 2.29)	0.74 (0.24, 2.29)	0.28	−0.44 (−1.78)	0.74 (0.24)
Pregnancy, puerperium and perinatal conditions	10036585	2	0.31 (0.08, 1.24)	0.31 (0.08, 1.25)	3.05	−1.68 (−2.97)	0.31 (0.08)
Social circumstances	10041244	2	0.24 (0.06, 0.97)	0.24 (0.06, 0.97)	4.71	−2.04 (−3.29)	0.24 (0.06)

Note: Ranked by case reports.

### Signal detection at PT level tested by four methods

The results of signal strength of AEs at the PT level tested positive by four methods ranked by ROR and repots were included in Supplementary Tables S2 and S3, respectively.

### Top 36 frequency of AEs at the PT level ranked by ROR

**FIGURE 3:**
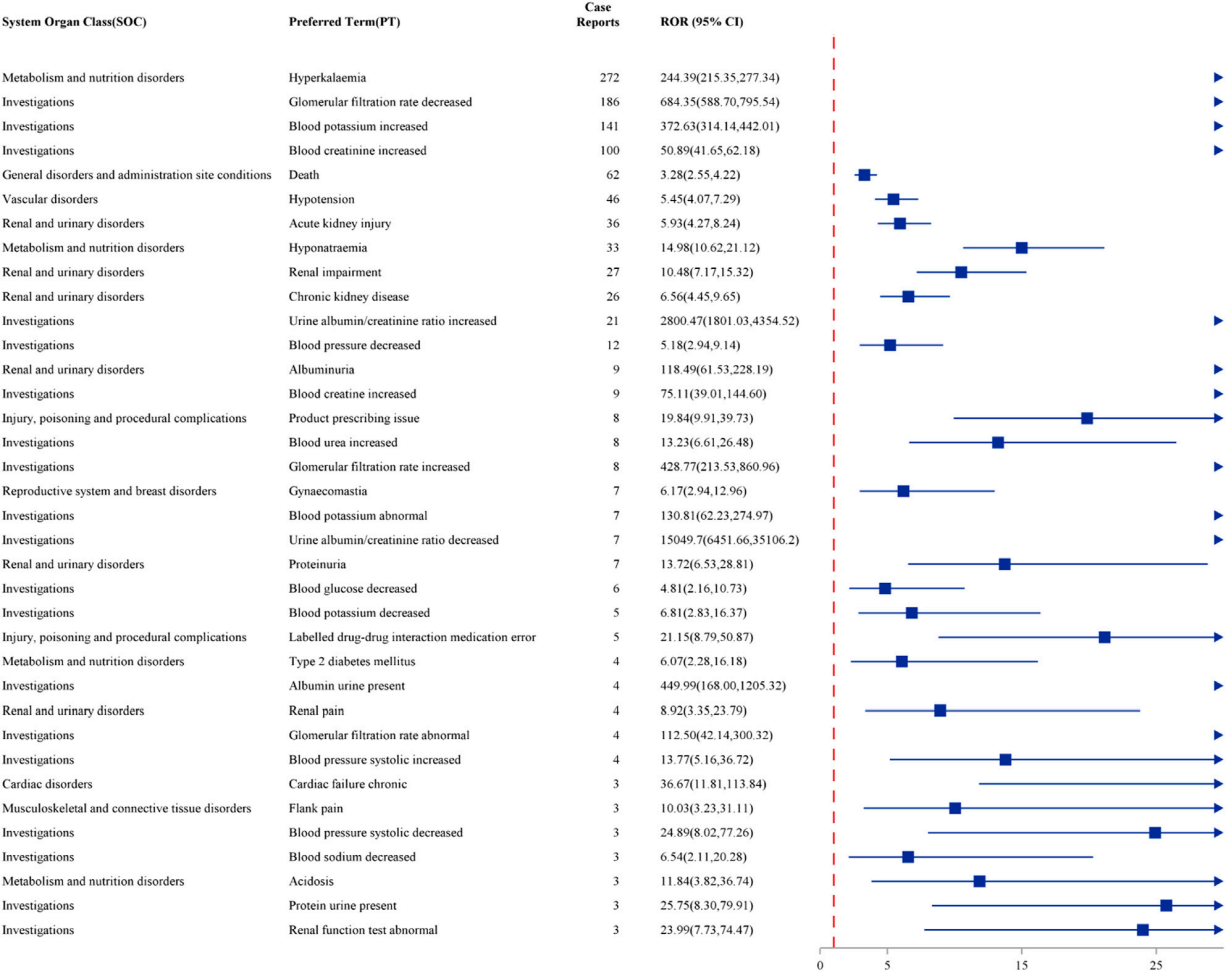
Forest plot of the top 36 most frequent AEs associated with finerenone usage at the PT level, ranked by ROR.

After a combination of PRR, ROR, PRR, BCPNN, and MGPS were utilized, Top 36 frequency of AEs tested consistently positive by four methods at the PT level was illustrated in Figure 3. The top ten frequency commonly reported AEs associated with PT are as follows: hyperkalaemia (N = 272, ROR = 244.39), glomerular filtration rate dereased (N = 186, ROR = 684.34), blood potassium increased (N = 141, ROR = 372.63), blood creatinine increased (N = 100, ROR = 50.89), death (N = 62, ROR = 3.28), hypotension (N = 46, ROR = 5.45), acute kidney injury (N = 36, ROR = 5.39), hyponatraemia (N = 33, ROR = 14.98), renal impairment (N = 27, ROR = 10.48) and chronic kidney disease (N = 26, ROR = 6.56).

### Top 36 strength of AEs at the PT level ranked by ROR

**FIGURE 4:**
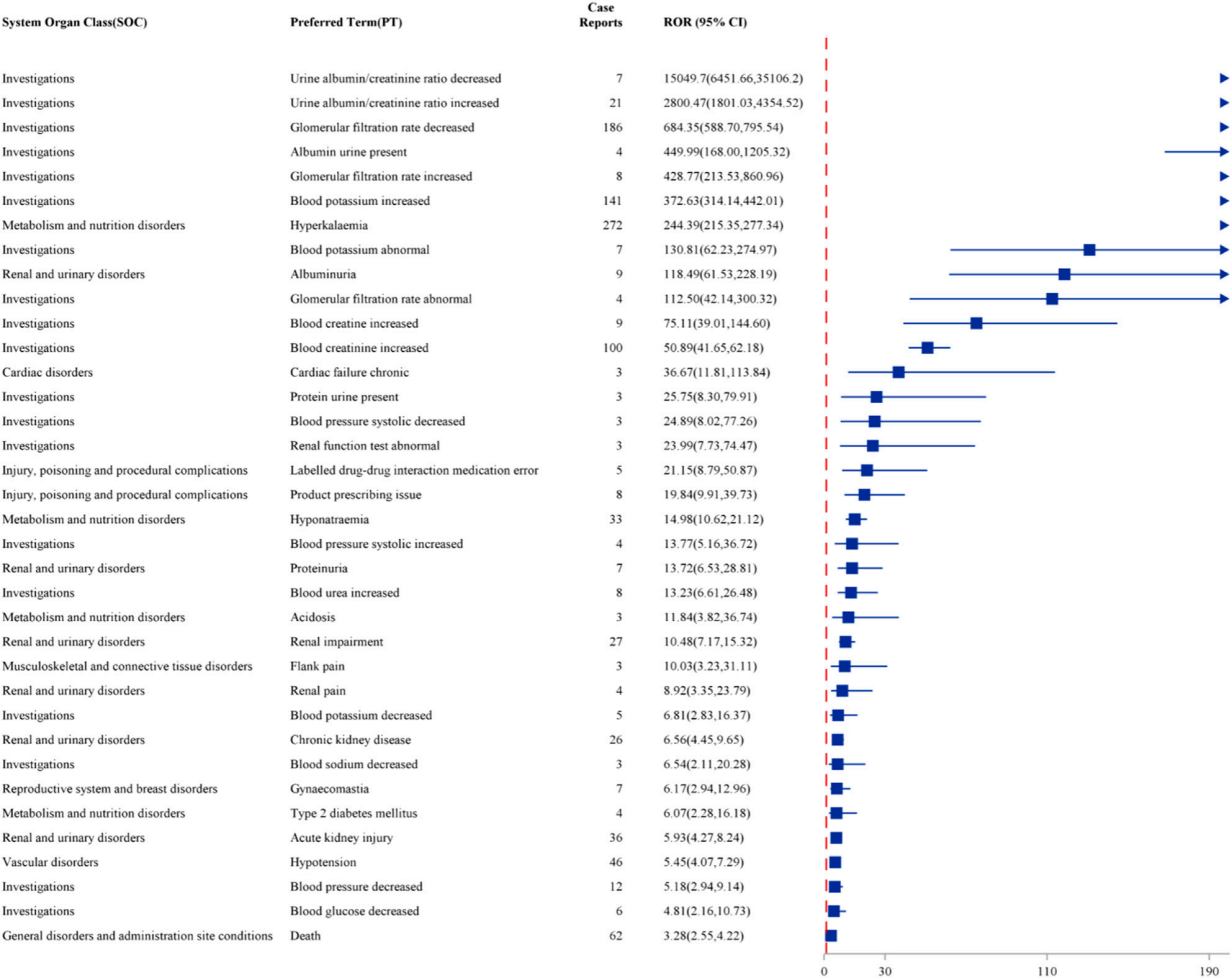
Forest plot of the top 36 strongest AEs associated with finerenone usage at the PT level, ranked by ROR.

After a combination of PRR, ROR, PRR, BCPNN, and MGPS were utilized, Top 36 strength of AEs tested consistently positive by four methods at the PT level was illustrated in Figure 4. The top ten strength commonly reported AEs associated with PT are as follows: urine albumin/creatinine ratio decreased (N = 7, ROR = 15049.7), urine albumin/creatinine ratio increased (N = 21, ROR = 2800.47), glomerular filtration rate decreased (N = 186, ROR = 684.35), albumin urine present (N = 4, ROR = 449.99), glomerular filtration rate increased (N = 8, ROR = 428.77), blood potassium increased (N = 141, ROR = 372.63), hyperkalaemia (N = 272, ROR = 244.39), blood potassium abnormal (N = 7, ROR = 130.81), albuminuria (N = 9, ROR = 118.49) and glomerular filtration rate abnormal (N = 4, ROR = 112.50).

## Discussion

In the course of our pharmacovigilance examination into finerenone, we discovered substantial adverse reaction patterns, notably in relation to the levels of potassium in the blood and the function of the kidneys. For the purpose of improving clinical application and the long-term prognosis of patients, it is essential to have a thorough understanding of these undesirable consequences.

The non-steroidal MRA finerenone is a novel medication that has been approved for use in the treatment of patients who suffer from type 2 diabetes and chronic renal disease. By inhibiting the activity of mineralocorticoid receptors, it reduces the amount of sodium that is reabsorption and potassium that is excreted, so reducing the strain on the cardiovascular system and the kidneys (Georgianos and Agarwal, 2023). While it is possible that it could be used to treat diabetic nephropathy, it is important to take into consideration the impact that it has on the equilibrium of electrolytes, particularly potassium levels (Wanner et al., 2022).

Based on an analysis of the WHO-VigiAccess database, it has been seen that the number of occurrences has been increasing on an annual basis from the first documentation of a finerenone-associated AE in the year 2015, with a significant increase occurring after the year 2022. The observed adverse consequences by the year 2024 included, but were not limited to, hyperkalemia, decreased glomerular filtration rate, increased blood potassium, and higher serum creatinine levels (Filippatos et al., 2022b). However, these were not the only unfavorable effects. Hyperkalemia is considered to be one of the adverse outcomes that is considered to be the most clinically significant. Due to the fact that it can lead to severe cardiovascular difficulties and even death, this illness is not only common but also exceedingly dangerous (Pitt et al., 2021).

According to the findings of our investigation, hyperkalemia was shown to be one of the most common and severe cases (Montford and Linas, 2017). On a fundamental level, this is connected to the mechanism of action of non-steroidal MRAs. This is because the inhibition of the mineralocorticoid receptor prevents sodium exchange and reduces potassium excretion, which ultimately leads to a significant accumulation of potassium ions in situations where renal function is compromised (Hundemer and Sood, 2021). Significant hyperkalemia can lead to heart arrhythmias, which is a potentially fatal outcome of the condition (Weiss et al., 2017). For example, excessive amounts of potassium in the blood can disrupt the normal electrical activity of cardiac muscle cells, which can lead to potentially fatal arrhythmias. As a consequence of this, it is of the utmost importance to carefully monitor the levels of potassium in the blood when undertaking MRAs therapy, particularly in patients who have a previous history of renal failure (Banerjee et al., 2021).

Owing to finerenone’s predictable effects on potassium homeostasis and renal function, clinicians should maintain heightened vigilance for adverse reactions-especially, though not exclusively, during the first few weeks of treatment (Rossing et al., 2022). It is vital to do routine evaluations of serum potassium levels and renal function indices in order to take preventative measures against severe adverse effects. Individuals who have a history of cardiovascular disease or renal insufficiency may require an adjustment of their dosage or the investigation of alternative therapeutic alternatives, depending on the results of the risk assessment (Kobayashi et al., 2024). In the pooled analysis of previous trials (Bakris et al., 2020; Pitt et al., 2021), patients with pre-existing CVD exhibited a significantly higher incidence of hyperkalaemia (serum K^+^ > 5.5 mmol L^−1^), necessitating protocol-defined dose reduction or temporary discontinuation of finerenone. Consequently, the 2024 KDIGO Clinical Practice Guideline and the ADA consensus recommend initiating finerenone at 10 mg once daily in CKD patients with concomitant CVD or heart failure, provided baseline serum K^+^ is ≤ 4.8 mmol L^−1^; escalation to 20 mg is permitted only if serum K^+^ remains ≤ 4.8 mmol L^−1^ after 4 weeks of treatment (Kidney Disease: Improving Global Outcomes KDIGO CKD Work Group, 2024).

The further study should concentrate on conducting extensive clinical studies in order to precisely evaluate the risk of AEs that are associated with finerenone and the dose relationship between the two (Chung et al., 2020). In the future, there will be advancements in technology and safety evaluations in the field of drug creation. Additionally, there will be an effort to immediately address new therapeutic challenges while simultaneously ensuring the safety and effectiveness of existing medicines (Fusaroli et al., 2024). When medication-related AEs are the focus of attention, drug safety standards are improved, and essential information is gathered that can be used to modify treatment plans and make improvements to healthcare delivery (Yang et al., 2023). In a society that is becoming increasingly globalized and networked, it is very necessary to improve pharmaceutical development and administration in order to guarantee the health and safety of the general public (Henderson et al., 2024). Through the promotion of inter-disciplinary collaboration and the implementation of continuous monitoring, we have the potential to improve our preparedness for public health emergencies and concerns regarding chronic illness, thereby protecting the health of individuals (Faksova et al., 2024).

In light of these considerations, nations are obligated to take an active part in primary drug safety research, with a particular emphasis on cohort event monitoring, in order to precisely establish the causal connections between drugs and AEs, like COVID-19 vaccines (Raethke et al., 2023). The formulation of more precise public health policies and the expansion of treatment programs will be made easier as a result of this, which will ensure that drugs like finerenone are both safe and effective (Götzinger et al., 2024). As a result of our analysis, which utilized a demanding signal detection approach, we have found specific adverse effects that require extra care in the therapeutic application of finerenone, particularly with regard to electrolyte balance and renal function. The occurrence of AEs highlights the necessity for enhanced patient monitoring and tailored treatment approaches in order to guarantee the safe and effective application of finerenone in clinical settings.

## Conclusion

The study revealed that 1,482 finerenone-related AEs were spontaneously reported in the VigiAccess database by November 2024. Among these, the most frequently reported symptoms included hyperkalaemia, decreased eGFR, and increased blood potassium levels. While the majority of AEs were minor and self-limiting, some serious AEs could lead to hospitalization and, in rare instances, death. It is crucial for healthcare systems to proactively engage in comprehensive safety studies, such as cohort event monitoring, to better establish causal relationships between AEs and finerenone use. Clinicians should closely monitor blood potassium levels and kidney function in patients receiving finerenone, making timely adjustments as necessary to ensure safe and effective treatment.

## Data Availability

The original contributions presented in the study are included in the article/Supplementary Material, further inquiries can be directed to the corresponding author.
