# Supplementary material for: Characteristic analysis of adverse reactions of finerenone: an in-depth analysis from WHO-VigiAccess
Source: Front Pharmacol. 2025 Aug 7;16:1545148. doi: 10.3389/fphar.2025.1545148 (PMC12367681; doi:10.3389/fphar.2025.1545148)
Supplement: Supplementary file 1 [file Table1.docx]

**Supplementary 1**

**Statistical analysis reports of adverse reactions mining based on the Vigiaccess database**

Introduction

In this study, we used disproportionate analyses ADR signals included in the Vigiaccess database.The study aimed to detect new and unexpected ADRs not described in the drug label^1^.

# Methods


## Data analysis

Two-by-two contingency table for disproportionality analysis.

| **Item** | **Target adverse events reported** | **Other adverse events reported** | **Total** |
| --- | --- | --- | --- |
| **Target drugs** | a | b | a + b |
| **Other drugs** | c | d | c + d |
| **Total** | a + c | b + d | a + b + c + d |

The principles of disproportionate measurement and the criteria for signal detection.

| Method | Calculation formula | ﻿Criteria |
| --- | --- | --- |
| ROR | $ROR=\frac{a / c}{b / d}$ | a ≥ 3  ROR ≥ 1  95%CI (lower limit) > 1 |
|  | $SE(lnROR)=\sqrt{\frac{1}{a}+\frac{1}{b}+\frac{1}{c}+\frac{1}{d}}$ |  |
|  | $95\%CI= e^{\ln\left( ROR \right)\pm1.96se}$ |  |
| PRR | $PRR=\frac{a / (a+b)}{c / (c+d)}$ | a ≥ 3  PRR ≥ 2  95%CI (lower limit) > 1 |
|  | $SE(lnPRR)=\sqrt{\frac{1}{a}-\frac{1}{a+b}+\frac{1}{c}-\frac{1}{c+d}}$ |  |
|  | $95\%CI= e^{\ln\left( PRR \right)\pm1.96se}$ |  |
|  | $\chi2 =\frac{{(ad-bc)}^{2}(a+b+c+d)}{( a+b)(a+c)(c+d)(b+d)}$ | a ≥ 3  PRR ≥ 2  $\chi2\geq4$ |
| BCPNN | IC=${log}_{2}\frac{p(x,y)}{p(x)p(y)}={log}_{2}\frac{a(a+b+c+d)}{(a+b)(a+c)}$ | IC025>0 |
|  | E(IC)=${log}_{2}\frac{(a+\gamma11)(a+b+c+d+\alpha)(a+b+c+d+\beta)}{（a+b+c+d+\gamma）(a+b+\alpha1)(a+c+\beta1)}$ |  |
|  | $V\left( IC \right)=\frac{1}{{(ln2)}^{2}}\{\left[ \frac{\left( a+b+c+d \right)-a+\gamma-\gamma11}{\left( a+\gamma11 \right)\left( 1+a+b+c+d+\gamma\right)} \right]+\left[ \frac{\left( a+b+c+d \right)-\left( a+b \right)+\alpha-\alpha1}{\left( a+b+\alpha1 \right)\left( 1+a+b+c+d+\alpha\right)} \right]+\left[ \frac{\left( a+b+c+d \right)-\left( a+c \right)+\beta-\beta1}{\left( a+c+\beta1 \right)\left( 1+a+b+c+d+\beta\right)} \right]\}$ |  |
|  | $\gamma=\gamma11\frac{(a+b+c+d+\alpha)(a+b+c+d+\beta)}{(a+b+\alpha1)(a+c+\beta1)}$ |  |
|  | *IC-2SD=E(IC)-2*$\sqrt{V(IC)}$  $\alpha1=\beta1=1；\alpha=\beta=2；\gamma11=1$ |  |
| EBGM | $EBGM=\frac{a(a+b+c+d)}{\left( a+c \right)(a+b)}$ | EBGM05>2 |
|  | $SE(lnEBGM)=\sqrt{\frac{1}{a}+\frac{1}{b}+\frac{1}{c}+\frac{1}{d}}$ |  |
|  | $95\%CI= e^{\ln\left( EBGM \right)\pm1.96se}$ |  |

# Other

**Calculatio definition of the disproportionality approach Bayesian information component**

$$\begin{aligned} IC=\log_{2}\left( \frac{N_{\mathrm{observed}}+ 0.5}{N_{\mathrm{expected}}+ 0.5} \right)\#\left( 1 \right) \end{aligned}$$

$$\begin{aligned} N_{\mathrm{expected}}=\frac{\left( N_{\mathrm{drug}}*N_{\mathrm{effect}} \right)}{N_{\mathrm{total}}}\#\left( 2 \right) \end{aligned}$$

$$\begin{aligned} \mathrm{IC}_{025}=\log_{2}\left( \frac{N_{\mathrm{observed}}+ 0.5}{N_{\mathrm{expected}}+ 0.5} \right)-3.3*\left( N_{\mathrm{observed}}+0.5 \right)^{-\frac{1}{2}}-2*\left( N_{\mathrm{observed}}+0.5 \right)^{-\frac{3}{2}}\#\left( 3 \right) \end{aligned}$$

$$\begin{aligned} \mathrm{IC}_{975}=\log_{2}\left( \frac{N_{\mathrm{observed}}+ 0.5}{N_{\mathrm{expected}}+ 0.5} \right)+2.4*\left( N_{\mathrm{observed}}+0.5 \right)^{-\frac{1}{2}}-0.5*\left( N_{\mathrm{observed}}+0.5 \right)^{-\frac{3}{2}}\#\left( 4 \right) \end{aligned}$$

N_expected_: the number of case reports expected for the drug-ADR pairs.

N_observed_: the actual number of case reports for the drug-ADR pairs.

N_effect_: the number of case reports for the ADR, regardless of the drug.

N_total_: the total number of case reports in the database.

N_drug_: the number of case reports for the drug, regardless of the ADR.

**Reference:**

1. Norén GN, Hopstadius J, Bate A. Shrinkage observed-to-expected ratios for robust and transparent large-scale pattern discovery. Stat Methods Med Res 2013;22:57-69.

**Supplementary 2**

Table Signal strength of adverse events at the Preferred Term(PT) level ranked by Reports

| **System Organ Class(SOC)** | **Preferred Term(PT)** | **Case reports** | **ROR (95% CI)** | **PRR (95% CI)** | **Chi Square** | **IC (IC025)** | **EBGM (EBGM05)** |
| --- | --- | --- | --- | --- | --- | --- | --- |
| Metabolism and nutrition disorders | Hyperkalaemia | 272 | 244.39  (215.35,277.34) | 216.63  (193.65,242.34) | 58155.8 | 7.75  (6.73) | 215.69  (190.06) |
| Investigations | Glomerular filtration rate decreased | 186 | 684.35  (588.70,795.54) | 631.06  (549.19,725.13) | 115541 | 9.28  (6.95) | 623.09  (536.01) |
| Investigations | Blood potassium increased | 141 | 372.63  (314.14,442.01) | 350.66  (298.60,411.80) | 48822.6 | 8.44  (6.41) | 348.19  (293.54) |
| Investigations | Blood creatinine increased | 100 | 50.89  (41.65,62.18) | 48.80  (40.28,59.12) | 4681.28 | 5.61  (4.76) | 48.75  (39.90) |
| General disorders and administration site conditions | Death | 62 | 3.28  (2.55,4.22) | 3.22  (2.52,4.12) | 95.62 | 1.69  (1.27) | 3.22  (2.50) |
| Vascular disorders | Hypotension | 46 | 5.45  (4.07,7.29) | 5.36  (4.03,7.14) | 163.74 | 2.42  (1.87) | 5.36  (4.00) |
| Renal and urinary disorders | Acute kidney injury | 36 | 5.93  (4.27,8.24) | 5.86  (4.23,8.10) | 145.34 | 2.55  (1.89) | 5.86  (4.21) |
| Metabolism and nutrition disorders | Hyponatraemia | 33 | 14.98  (10.62,21.12) | 14.79  (10.54,20.75) | 424.46 | 3.89  (2.90) | 14.78  (10.48) |
| Renal and urinary disorders | Renal impairment | 27 | 10.48  (7.17,15.32) | 10.38  (7.13,15.10) | 229.00 | 3.38  (2.41) | 10.38  (7.10) |
| Renal and urinary disorders | Chronic kidney disease | 26 | 6.56  (4.45,9.65) | 6.50  (4.43,9.52) | 121.09 | 2.70  (1.87) | 6.50  (4.41) |
| Investigations | Urine albumin/creatinine ratio increased | 21 | 2800.47  (1801.03,4354.52) | 2775.82  (1791.76,4300.33) | 55145.7 | 11.36  (3.81) | 2627.92  (1690.07) |
| Investigations | Blood pressure decreased | 12 | 5.18  (2.94,9.14) | 5.16  (2.94,9.08) | 40.31 | 2.37  (1.16) | 5.16  (2.93) |
| Renal and urinary disorders | Albuminuria | 9 | 118.49  (61.53,228.19) | 118.05  (61.45,226.77) | 1042.03 | 6.88  (2.30) | 117.77  (61.15) |
| Investigations | Blood creatine increased | 9 | 75.11  (39.01,144.60) | 74.83  (38.96,143.71) | 654.62 | 6.22  (2.24) | 74.72  (38.81) |
| Injury, poisoning and procedural complications | Product prescribing issue | 8 | 19.84  (9.91,39.73) | 19.78  (9.90,39.51) | 142.59 | 4.31  (1.72) | 19.77  (9.87) |
| Investigations | Blood urea increased | 8 | 13.23  (6.61,26.48) | 13.18  (6.60,26.34) | 90.08 | 3.72  (1.52) | 13.18  (6.58) |
| Investigations | Glomerular filtration rate increased | 8 | 428.77  (213.53,860.96) | 427.34  (213.31,856.09) | 3373.49 | 8.73  (2.17) | 423.67  (211.00) |
| Reproductive system and breast disorders | Gynaecomastia | 7 | 6.17  (2.94,12.96) | 6.16  (2.94,12.90) | 30.26 | 2.62  (0.88) | 6.16  (2.93) |
| Investigations | Blood potassium abnormal | 7 | 130.81  (62.23,274.97) | 130.43  (62.19,273.57) | 896.74 | 7.02  (1.90) | 130.09  (61.89) |
| Investigations | Urine albumin/creatinine ratio decreased | 7 | 15049.7  (6451.66,35106.2) | 15005.5  (6445.00,34936.4) | 80518.9 | 13.49  (1.85) | 11504.5  (4931.86) |
| Renal and urinary disorders | Proteinuria | 7 | 13.72  (6.53,28.81) | 13.68  (6.53,28.67) | 82.28 | 3.77  (1.38) | 13.68  (6.51) |
| Investigations | Blood glucose decreased | 6 | 4.81  (2.16,10.73) | 4.81  (2.16,10.69) | 18.09 | 2.26  (0.55) | 4.80  (2.16) |
| Investigations | Blood potassium decreased | 5 | 6.81  (2.83,16.37) | 6.80  (2.83,16.31) | 24.72 | 2.76  (0.61) | 6.80  (2.83) |
| Injury, poisoning and procedural complications | Labelled drug-drug interaction medication error | 5 | 21.15  (8.79,50.87) | 21.11  (8.79,50.68) | 95.74 | 4.40  (1.10) | 21.10  (8.77) |
| Metabolism and nutrition disorders | Type 2 diabetes mellitus | 4 | 6.07  (2.28,16.18) | 6.06  (2.28,16.13) | 16.90 | 2.60  (0.30) | 6.06  (2.27) |
| Investigations | Albumin urine present | 4 | 449.99  (168.00,1205.32) | 449.24  (167.99,1201.33) | 1772.81 | 8.80  (1.01) | 445.19  (166.21) |
| Renal and urinary disorders | Renal pain | 4 | 8.92  (3.35,23.79) | 8.91  (3.35,23.72) | 28.08 | 3.15  (0.49) | 8.91  (3.34) |
| Investigations | Glomerular filtration rate abnormal | 4 | 112.50  (42.14,300.32) | 112.31  (42.14,299.33) | 440.28 | 6.81  (0.98) | 112.06  (41.97) |
| Investigations | Blood pressure systolic increased | 4 | 13.77  (5.16,36.72) | 13.75  (5.16,36.60) | 47.26 | 3.78  (0.66) | 13.74  (5.15) |
| Cardiac disorders | Cardiac failure chronic | 3 | 36.67  (11.81,113.84) | 36.63  (11.82,113.54) | 103.90 | 5.19  (0.44) | 36.60  (11.79) |
| Musculoskeletal and connective tissue disorders | Flank pain | 3 | 10.03  (3.23,31.11) | 10.01  (3.23,31.03) | 24.34 | 3.32  (0.18) | 10.01  (3.23) |
| Investigations | Blood pressure systolic decreased | 3 | 24.89  (8.02,77.26) | 24.86  (8.02,77.06) | 68.68 | 4.64  (0.39) | 24.85  (8.01) |
| Investigations | Blood sodium decreased | 3 | 6.54  (2.11,20.28) | 6.53  (2.11,20.23) | 14.05 | 2.71  (0.01) | 6.53  (2.10) |
| Metabolism and nutrition disorders | Acidosis | 3 | 11.84  (3.82,36.74) | 11.83  (3.82,36.64) | 29.73 | 3.56  (0.23) | 11.82  (3.81) |
| Investigations | Protein urine present | 3 | 25.75  (8.30,79.91) | 25.71  (8.30,79.70) | 71.23 | 4.68  (0.40) | 25.70  (8.28) |
| Investigations | Renal function test abnormal | 3 | 23.99  (7.73,74.47) | 23.96  (7.73,74.27) | 65.99 | 4.58  (0.39) | 23.95  (7.72) |

Note1:ranked by Reports

Note2:Signals are detected when all the following criteria are met:a ≥ 3, PRR ≥2 and Chi-Square ≥ 4, lower limit of 95% CI of ROR > 1, IC025 > 0, EBGM05 > 2.

**Supplementary 3**

Table Signal strength of adverse events at the Preferred Term(PT) level ranked by ROR

| **System Organ Class(SOC)** | **Preferred Term(PT)** | **Case reports** | **ROR (95% CI)** | **PRR (95% CI)** | **Chi Square** | **IC (IC025)** | **EBGM (EBGM05)** |
| --- | --- | --- | --- | --- | --- | --- | --- |
| Investigations | Urine albumin/creatinine ratio decreased | 7 | 15049.7  (6451.66,35106.2) | 15005.5  (6445.00,34936.4) | 80518.9 | 13.49  (1.85) | 11504.5  (4931.86) |
| Investigations | Urine albumin/creatinine ratio increased | 21 | 2800.47  (1801.03,4354.52) | 2775.82  (1791.76,4300.33) | 55145.7 | 11.36  (3.81) | 2627.92  (1690.07) |
| Investigations | Glomerular filtration rate decreased | 186 | 684.35  (588.70,795.54) | 631.06  (549.19,725.13) | 115541 | 9.28  (6.95) | 623.09  (536.01) |
| Investigations | Albumin urine present | 4 | 449.99  (168.00,1205.32) | 449.24  (167.99,1201.33) | 1772.81 | 8.80  (1.01) | 445.19  (166.21) |
| Investigations | Glomerular filtration rate increased | 8 | 428.77  (213.53,860.96) | 427.34  (213.31,856.09) | 3373.49 | 8.73  (2.17) | 423.67  (211.00) |
| Investigations | Blood potassium increased | 141 | 372.63  (314.14,442.01) | 350.66  (298.60,411.80) | 48822.6 | 8.44  (6.41) | 348.19  (293.54) |
| Metabolism and nutrition disorders | Hyperkalaemia | 272 | 244.39  (215.35,277.34) | 216.63  (193.65,242.34) | 58155.8 | 7.75  (6.73) | 215.69  (190.06) |
| Investigations | Blood potassium abnormal | 7 | 130.81  (62.23,274.97) | 130.43  (62.19,273.57) | 896.74 | 7.02  (1.90) | 130.09  (61.89) |
| Renal and urinary disorders | Albuminuria | 9 | 118.49  (61.53,228.19) | 118.05  (61.45,226.77) | 1042.03 | 6.88  (2.30) | 117.77  (61.15) |
| Investigations | Glomerular filtration rate abnormal | 4 | 112.50  (42.14,300.32) | 112.31  (42.14,299.33) | 440.28 | 6.81  (0.98) | 112.06  (41.97) |
| Investigations | Blood creatine increased | 9 | 75.11  (39.01,144.60) | 74.83  (38.96,143.71) | 654.62 | 6.22  (2.24) | 74.72  (38.81) |
| Investigations | Blood creatinine increased | 100 | 50.89  (41.65,62.18) | 48.80  (40.28,59.12) | 4681.28 | 5.61  (4.76) | 48.75  (39.90) |
| Cardiac disorders | Cardiac failure chronic | 3 | 36.67  (11.81,113.84) | 36.63  (11.82,113.54) | 103.90 | 5.19  (0.44) | 36.60  (11.79) |
| Investigations | Protein urine present | 3 | 25.75  (8.30,79.91) | 25.71  (8.30,79.70) | 71.23 | 4.68  (0.40) | 25.70  (8.28) |
| Investigations | Blood pressure systolic decreased | 3 | 24.89  (8.02,77.26) | 24.86  (8.02,77.06) | 68.68 | 4.64  (0.39) | 24.85  (8.01) |
| Investigations | Renal function test abnormal | 3 | 23.99  (7.73,74.47) | 23.96  (7.73,74.27) | 65.99 | 4.58  (0.39) | 23.95  (7.72) |
| Injury, poisoning and procedural complications | Labelled drug-drug interaction medication error | 5 | 21.15  (8.79,50.87) | 21.11  (8.79,50.68) | 95.74 | 4.40  (1.10) | 21.10  (8.77) |
| Injury, poisoning and procedural complications | Product prescribing issue | 8 | 19.84  (9.91,39.73) | 19.78  (9.90,39.51) | 142.59 | 4.31  (1.72) | 19.77  (9.87) |
| Metabolism and nutrition disorders | Hyponatraemia | 33 | 14.98  (10.62,21.12) | 14.79  (10.54,20.75) | 424.46 | 3.89  (2.90) | 14.78  (10.48) |
| Investigations | Blood pressure systolic increased | 4 | 13.77  (5.16,36.72) | 13.75  (5.16,36.60) | 47.26 | 3.78  (0.66) | 13.74  (5.15) |
| Renal and urinary disorders | Proteinuria | 7 | 13.72  (6.53,28.81) | 13.68  (6.53,28.67) | 82.28 | 3.77  (1.38) | 13.68  (6.51) |
| Investigations | Blood urea increased | 8 | 13.23  (6.61,26.48) | 13.18  (6.60,26.34) | 90.08 | 3.72  (1.52) | 13.18  (6.58) |
| Metabolism and nutrition disorders | Acidosis | 3 | 11.84  (3.82,36.74) | 11.83  (3.82,36.64) | 29.73 | 3.56  (0.23) | 11.82  (3.81) |
| Renal and urinary disorders | Renal impairment | 27 | 10.48  (7.17,15.32) | 10.38  (7.13,15.10) | 229.00 | 3.38  (2.41) | 10.38  (7.10) |
| Musculoskeletal and connective tissue disorders | Flank pain | 3 | 10.03  (3.23,31.11) | 10.01  (3.23,31.03) | 24.34 | 3.32  (0.18) | 10.01  (3.23) |
| Renal and urinary disorders | Renal pain | 4 | 8.92  (3.35,23.79) | 8.91  (3.35,23.72) | 28.08 | 3.15  (0.49) | 8.91  (3.34) |
| Investigations | Blood potassium decreased | 5 | 6.81  (2.83,16.37) | 6.80  (2.83,16.31) | 24.72 | 2.76  (0.61) | 6.80  (2.83) |
| Renal and urinary disorders | Chronic kidney disease | 26 | 6.56  (4.45,9.65) | 6.50  (4.43,9.52) | 121.09 | 2.70  (1.87) | 6.50  (4.41) |
| Investigations | Blood sodium decreased | 3 | 6.54  (2.11,20.28) | 6.53  (2.11,20.23) | 14.05 | 2.71  (0.01) | 6.53  (2.10) |
| Reproductive system and breast disorders | Gynaecomastia | 7 | 6.17  (2.94,12.96) | 6.16  (2.94,12.90) | 30.26 | 2.62  (0.88) | 6.16  (2.93) |
| Metabolism and nutrition disorders | Type 2 diabetes mellitus | 4 | 6.07  (2.28,16.18) | 6.06  (2.28,16.13) | 16.90 | 2.60  (0.30) | 6.06  (2.27) |
| Renal and urinary disorders | Acute kidney injury | 36 | 5.93  (4.27,8.24) | 5.86  (4.23,8.10) | 145.34 | 2.55  (1.89) | 5.86  (4.21) |
| Vascular disorders | Hypotension | 46 | 5.45  (4.07,7.29) | 5.36  (4.03,7.14) | 163.74 | 2.42  (1.87) | 5.36  (4.00) |
| Investigations | Blood pressure decreased | 12 | 5.18  (2.94,9.14) | 5.16  (2.94,9.08) | 40.31 | 2.37  (1.16) | 5.16  (2.93) |
| Investigations | Blood glucose decreased | 6 | 4.81  (2.16,10.73) | 4.81  (2.16,10.69) | 18.09 | 2.26  (0.55) | 4.80  (2.16) |
| General disorders and administration site conditions | Death | 62 | 3.28  (2.55,4.22) | 3.22  (2.52,4.12) | 95.62 | 1.69  (1.27) | 3.22  (2.50) |

Note1:ranked by ROR

Note2:Signals are detected when all the following criteria are met:a ≥ 3, PRR ≥2 and Chi-Square ≥ 4, lower limit of 95% CI of ROR > 1, IC025 > 0, EBGM05 > 2.
